# Supplementary material for: Opposing functions of the plant TOPLESS gene family during SNC1-mediated autoimmunity
Source: PLoS Genet. 2021 Feb 23;17(2):e1009026. doi: 10.1371/journal.pgen.1009026 (PMC7935258; doi:10.1371/journal.pgen.1009026)
Supplement: S1 Fig — Shoot weight from plants grown under short day conditions at 21°C for four weeks. Dots represent individual data points taken over two separate experiments. Whiskers on boxplots are drawn to the farthest data point within 1.5 * IQR of first and third quartiles. Letters denote significant differences as determined by Student’s t-test (P<0.01) using the Bonferroni-Holm method to correct for multiple comparisons. (PDF) [file pgen.1009026.s001.pdf]

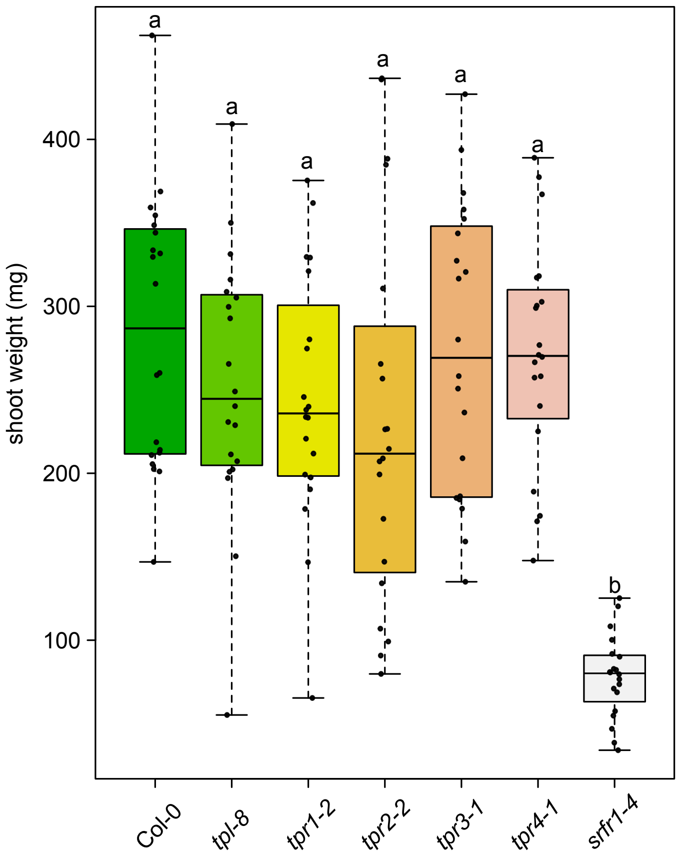

S1 Fig. Shoot weights of *tpl/tpr* single mutants do not differ significantly from Col-0

Shoot weight from plants grown under short day conditions at 21ºC for four weeks. Dots represent individual data points taken over two separate experiments. Whiskers on boxplots are drawn to the farthest data point within 1.5 * IQR of first and third quartiles. Letters denote significant differences as determined by Student's t-test (*P*<0.01) using the Bonferroni-Holm method to correct for multiple comparisons.
