# Supplementary material for: Opposing functions of the plant TOPLESS gene family during SNC1-mediated autoimmunity
Source: PLoS Genet. 2021 Feb 23;17(2):e1009026. doi: 10.1371/journal.pgen.1009026 (PMC7935258; doi:10.1371/journal.pgen.1009026)
Supplement: S2 Fig — Western blot of total protein extracted from srfr1-4, srfr1-4 tpl-8, srfr1-4 tpr1-2, srfr1-4 tpr2-2, srfr1-4 tpr3-1, and srfr1-4 tpr4-1. The large subunit of rubisco is shown as a loading control. (PDF) [file pgen.1009026.s002.pdf]

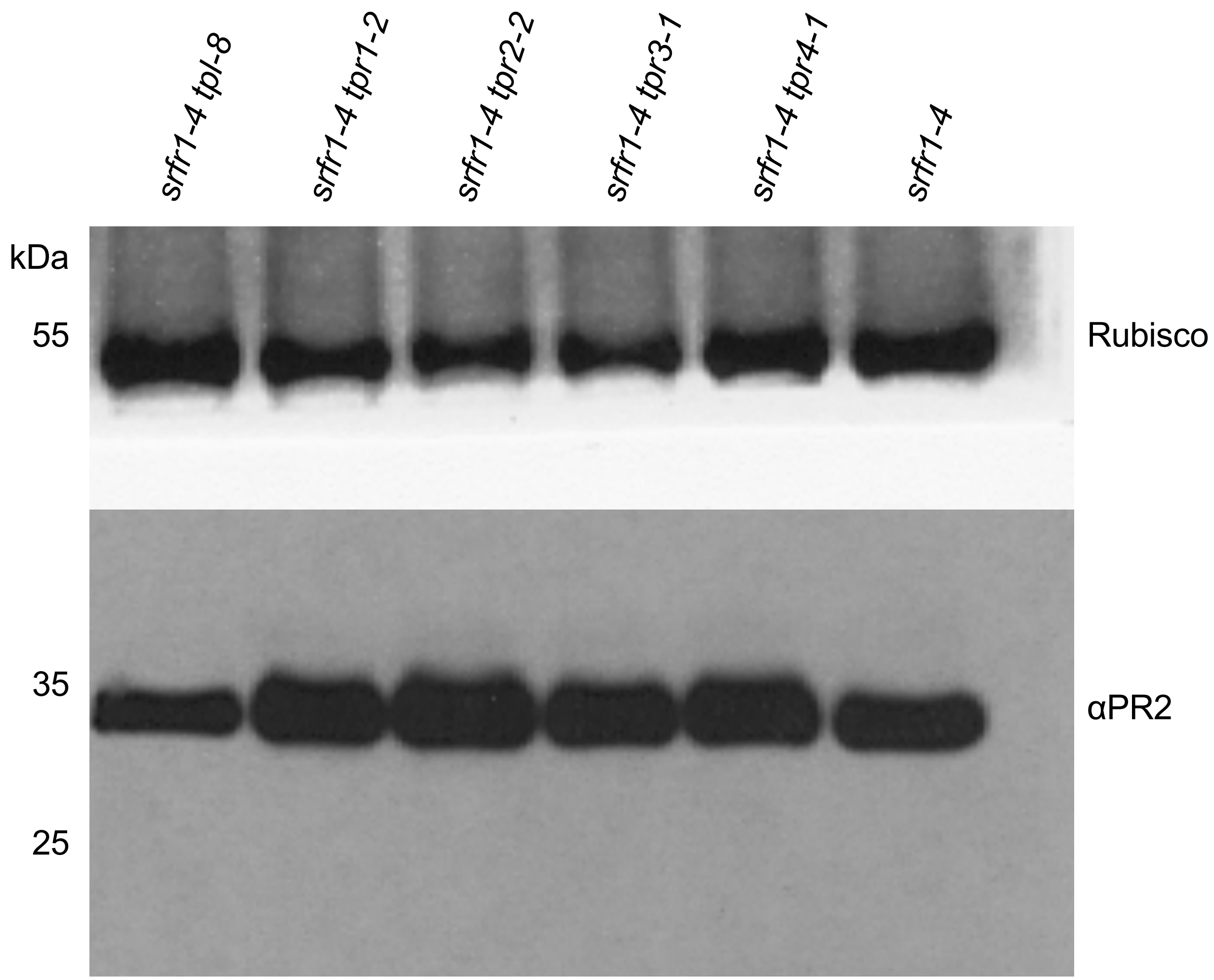

S2 Fig. PR2 expression in *srfr1-4* is affected by *tpl* and *tpr2*

Western blot of total protein extracted from *srfr1-4, srfr1-4 tpl-8*, *srfr1-4 tpr1-2*, *srfr1-4 tpr2-2*, *srfr1-4 tpr3-1*, and *srfr1-4 tpr4-1*. The large subunit of rubisco is shown as a loading control.
