# Supplementary material for: Opposing functions of the plant TOPLESS gene family during SNC1-mediated autoimmunity
Source: PLoS Genet. 2021 Feb 23;17(2):e1009026. doi: 10.1371/journal.pgen.1009026 (PMC7935258; doi:10.1371/journal.pgen.1009026)
Supplement: S3 Fig — Growth phenotype of srfr1-4 and srfr1-4 tpl/tpr double mutants at 280 (top row) and 21°C (bottom). (PDF) [file pgen.1009026.s003.pdf]

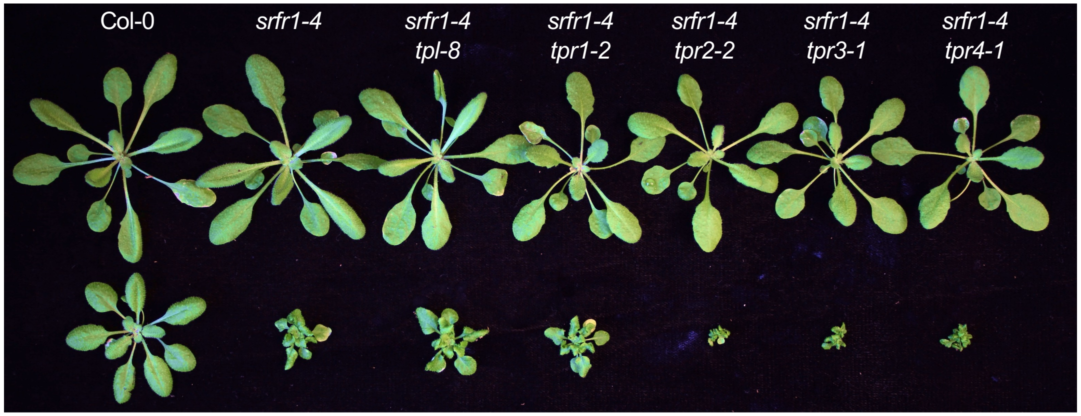

S3 Fig. Comparison of stunting in *srfr1-4 tpl/tpr* double mutants at 28º and 21ºC

Growth phenotype of *srfr1-4* and *srfr1-4 tpl/tpr* double mutants at 28º (top row) and 21ºC (bottom). Plants were grown at the indicated temperatures for 4 weeks under short-day conditions (8 h light / 16 h dark).
