# Supplementary material for: Opposing functions of the plant TOPLESS gene family during SNC1-mediated autoimmunity
Source: PLoS Genet. 2021 Feb 23;17(2):e1009026. doi: 10.1371/journal.pgen.1009026 (PMC7935258; doi:10.1371/journal.pgen.1009026)
Supplement: S4 Fig — (A) T-DNA insertion locations for tpr2-1 (SALK_112730) in exon 13, and tpr2-2 (SALK_079848) in exon 21. Scale bar is 200 bp. (B) RT-PCR using TPR2 primers on the 3’ side of the T-DNA insertions in tpr2-1 and tpr2-2 after 33 cycles. Actin was used as a control for quality of RNA and efficiency of reverse transcription. (PDF) [file pgen.1009026.s004.pdf]

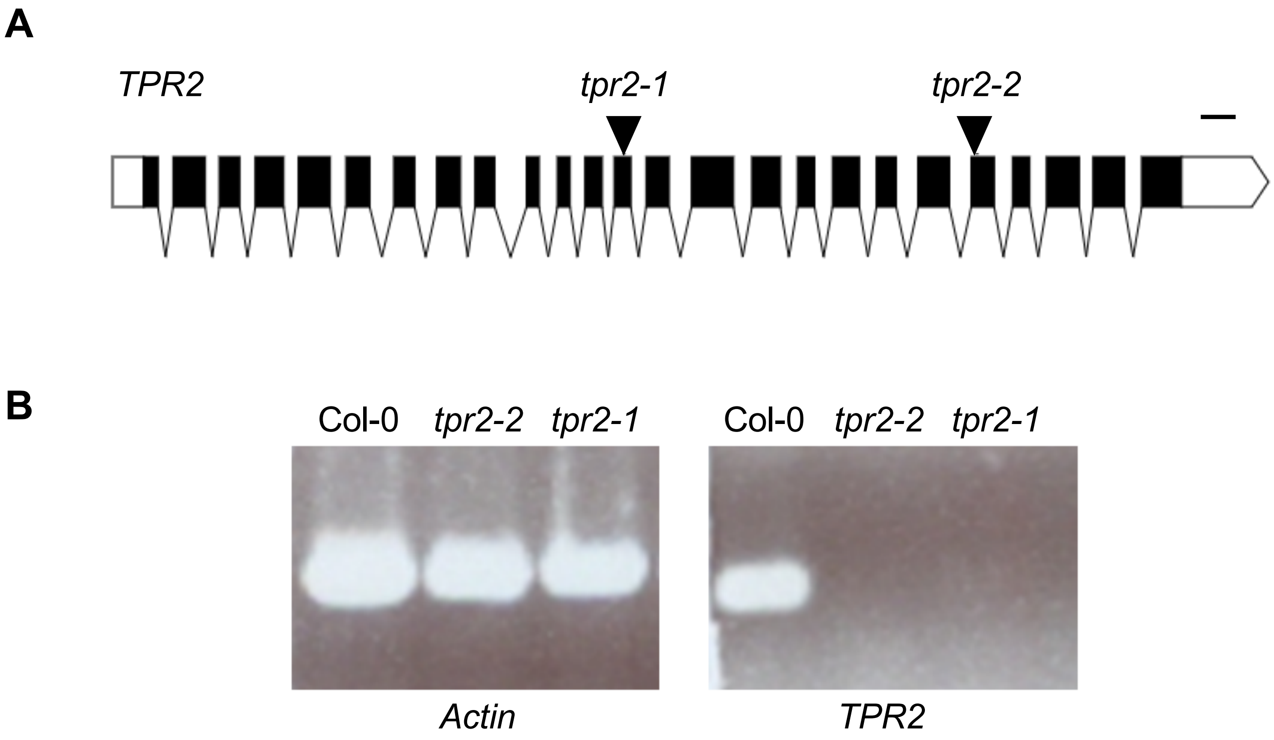

S4 Fig. Molecular characterization of *tpr2* T-DNA insertion alleles

(A) T-DNA insertion locations for *tpr2-1* (SALK_112730) in exon 13, and *tpr2-2* (SALK_079848) in exon 21. Scale bar is 200 bp. (B) RT-PCR using *TPR2* primers on the 3' side of the T-DNA insertions in *tpr2-1* and *tpr2-2* after 33 cycles. *Actin* was used as a control for quality of RNA and efficiency of reverse transcription. *TPR2* primers used were TPR2 cDNA For4 (S2 Table) in exon 21 and TPR2-2 LP (S1 Table) in exon 24.
