# Supplementary material for: Opposing functions of the plant TOPLESS gene family during SNC1-mediated autoimmunity
Source: PLoS Genet. 2021 Feb 23;17(2):e1009026. doi: 10.1371/journal.pgen.1009026 (PMC7935258; doi:10.1371/journal.pgen.1009026)
Supplement: S5 Fig — Phylogram showing evolutionary relationships amongst TOPLESS family members. The WD40 protein LEUNIG (LUG) is included as the outgroup. Tree was generated from full length cDNA sequences using www.phylogeny.fr. (PDF) [file pgen.1009026.s005.pdf]

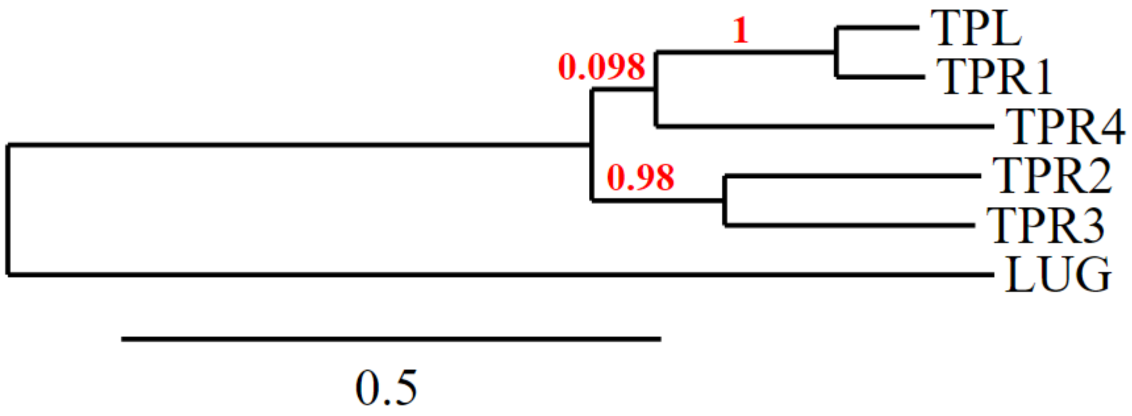

S5 Fig. Phylogenetic tree of the Arabidopsis TOPLESS family

Phylogram showing evolutionary relationships amongst *TOPLESS* family members. The WD40 protein *LEUNIG* (*LUG*) is included as the outgroup. Tree was generated from full length cDNA sequences using www.phylogeny.fr.
