# Supplementary material for: Opposing functions of the plant TOPLESS gene family during SNC1-mediated autoimmunity
Source: PLoS Genet. 2021 Feb 23;17(2):e1009026. doi: 10.1371/journal.pgen.1009026 (PMC7935258; doi:10.1371/journal.pgen.1009026)
Supplement: S6 Fig — In planta bacterial growth assay with the indicated plant genotypes and DC3000 infiltrated at a bacterial density of 5×10^4^ colony-forming units (cfu) per ml. Values represent averages from two independent experiments with triplicate samples, and error bars denote standard deviation. As determined by Student’s t-test with the Bonferroni-Holm method to correct for multiple comparisons, none of the values were significantly different with P≥0.2. (PDF) [file pgen.1009026.s006.pdf]

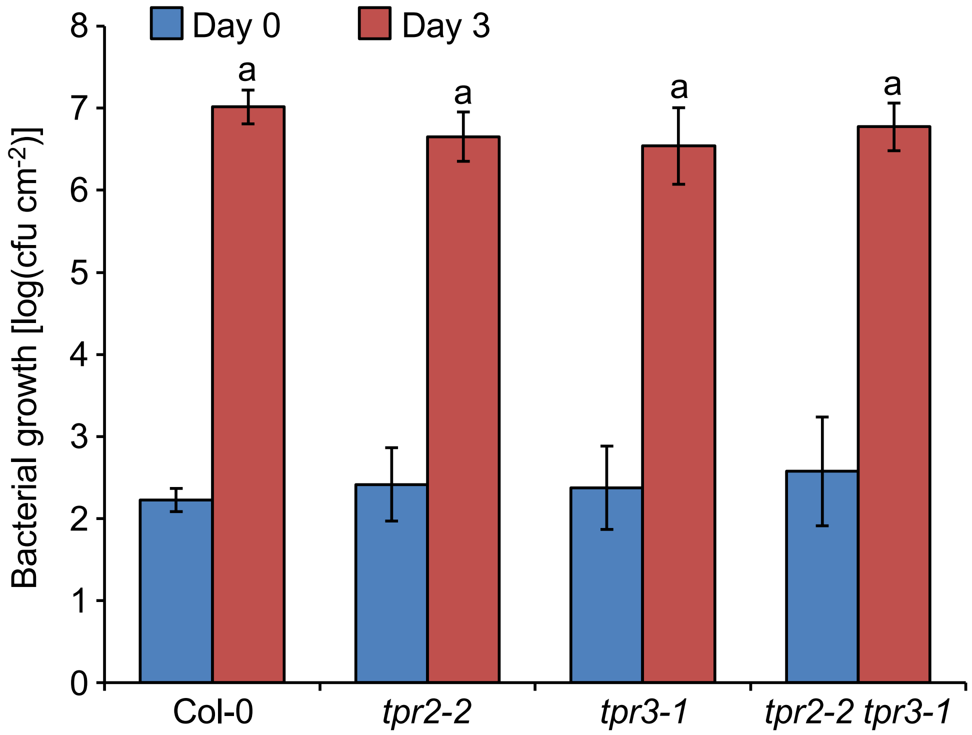

S6 Fig. Slightly elevated *SNC1* expression in *tpr2-2 tpr3-1* plants does not lead to increased bacterial resistance
In planta bacterial growth assay with the indicated plant genotypes and DC3000 infiltrated at a bacterial density of 5×10^4 colony-forming units (cfu) per ml. Values represent averages from two independent experiments with triplicate samples, and error bars denote standard deviation. As determined by Student's t-test with the Bonferroni-Holm method to correct for multiple comparisons, none of the values were significantly different with $P \geq 0.2$.
