# Supplementary material for: Opposing functions of the plant TOPLESS gene family during SNC1-mediated autoimmunity
Source: PLoS Genet. 2021 Feb 23;17(2):e1009026. doi: 10.1371/journal.pgen.1009026 (PMC7935258; doi:10.1371/journal.pgen.1009026)
Supplement: S7 Fig — In planta bacterial growth assay with the indicated plant genotypes and DC3000 infiltrated at a bacterial density of 5×10^4^ cfu/ml. Values represent averages from two independent experiments with triplicate samples, and error bars denote standard deviation. Letters denote statistically significant differences as determined by Student’s t-test with the Bonferroni-Holm method to correct for multiple comparisons (P<0.05). (PDF) [file pgen.1009026.s007.pdf]

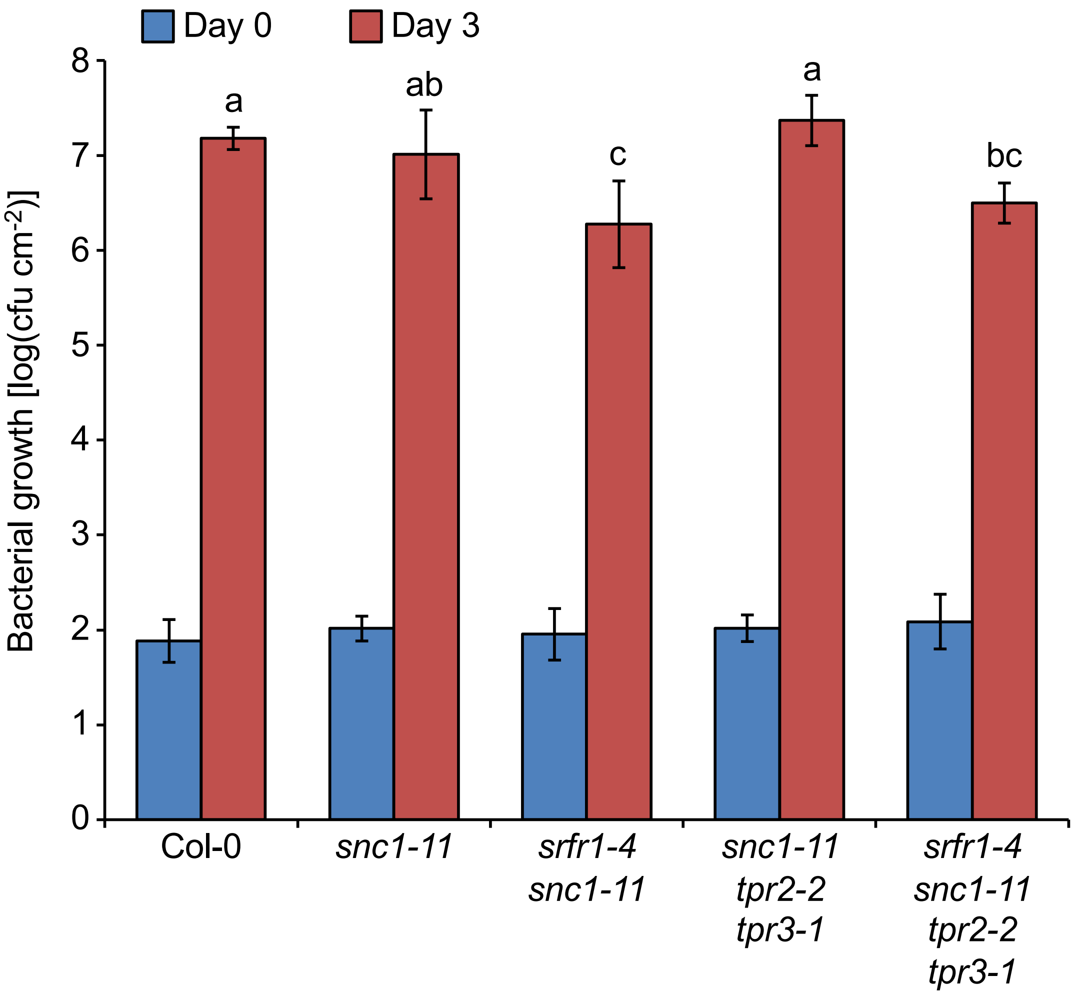

S7 Fig. *PR2* expression levels correlate with degree of bacterial resistance

In planta bacterial growth assay with the indicated plant genotypes and DC3000 infiltrated at a bacterial density of 5×10^4 cfu/ml. Values represent averages from two independent experiments with triplicate samples, and error bars denote standard deviation. Letters denote statistically significant differences as determined by Student's t-test with the Bonferroni-Holm method to correct for multiple comparisons *(P*<0.05).
