# Supplementary material for: Opposing functions of the plant TOPLESS gene family during SNC1-mediated autoimmunity
Source: PLoS Genet. 2021 Feb 23;17(2):e1009026. doi: 10.1371/journal.pgen.1009026 (PMC7935258; doi:10.1371/journal.pgen.1009026)
Supplement: S1 Table — (PDF) [file pgen.1009026.s008.pdf]

S1 Table. PCR primers used for genotyping mutant lines

Name	sequence	Use
LBa1	TGGTTCACGTAGTGGGCCATCG	SALK line border primer
TPL8 LP	TTGGTTCTCGCGAAAGATTAG	tpl-8
TPL8 RP	AGGAGAGAGCCTTCCTTGTTG	tpl-8
TPR1-2 LP	AAGGCCTCGAGATACTTCTGC	tpr1-2
TPR1-2 RP	ACTCCGTTATCCGTCACCTTC	tpr1-2
TPR2-2 LP	TCAGCATCAAAGACTGCAATG	tpr2-2
TPR2-2 RP	TGGGAAGGTGATTCGTTGTAC	tpr2-2
TPR2-1 LP	TCCTTGTTGAATCTCAATCGG	tpr2-1
TPR2-1 RP	ACGTCAACACCTCGAGGTATG	tpr2-1
TPR3-1LP	GTTCTCTTGCAGCCTCAATTG	tpr3-1
TPR3-1RP	TTCCCACAATGTGATTTCTCC	tpr3-1
TPR4-1 GTF	ATGTCGTCACTCAGCAGAGAACTC	tpr4-1
TPR4-1 GTR	GCAAAGCTGATGTTGCCAGTTCAA	tpr4-1
SNC1-11 LP	TCGGCATAACATCGTAAGAGC	snc1-11
SNC1-11 RP	CAAGCTTTCGTGGAGAAGATG	snc1-11
SNC1 FOR GT	GGCATGCGTAATCTGCAATATCTAG	snc1-1
SNC1 LESLEY REV	GAGGTACTCGAGAGATTCCAAGTTG	snc1-1
SNC1-1 GT FOR	GGCATGCGTAATCTGCAATATCTAa	snc1-1
37460-18	TCTCCACTGTACTAATTTCCCT	srfr1-4
37460-R	ACTAATTCCGCAACGTGCCT	srfr1-4
EDS1 F2	CCCTTTCTAGTTTCCTTGAGCTAAG	eds1-2
EDS1 R3	TCAGGTATCTGTTATTTCATCCATC	eds1-2
