# Supplementary material for: Opposing functions of the plant TOPLESS gene family during SNC1-mediated autoimmunity
Source: PLoS Genet. 2021 Feb 23;17(2):e1009026. doi: 10.1371/journal.pgen.1009026 (PMC7935258; doi:10.1371/journal.pgen.1009026)
Supplement: S2 Table — (PDF) [file pgen.1009026.s009.pdf]

S2 Table. Primers used for qPCR

Name	sequence	Use
SAND CDNA FOR 1	CACTTGCAGACAAGGCGATG	qPCR
SAND CDNA REV 1	CCTTTGGCACACCTGATTGC	qPCR
TPR2 CDNA FOR4	ATTATTGCAATCGGGATGGA	qPCR
TPR2 CDNA RP5	CTTGGGGCACCCACTTATGA	qPCR
QRT SNC1 FOR1	GCGGTGTACGACTCATGTATGTC	qPCR
QRT SNC1 REV1	GATGTCATCCGCATCCGCTT	qPCR
PR2 QRT FOR1	TTCAACCACACAGCTGGACA	qPCR
PR2 QRT REV1	GGCAAGGTATCGCCTAGCAT	qPCR
RPP4 QRT FOR1	GGAAGGCATCCAGTCGCTT	qPCR
RPP4 QRT REV1	CACCAAACTTTTGCACCCGT	qPCR
