# Supplementary material for: Opposing functions of the plant TOPLESS gene family during SNC1-mediated autoimmunity
Source: PLoS Genet. 2021 Feb 23;17(2):e1009026. doi: 10.1371/journal.pgen.1009026 (PMC7935258; doi:10.1371/journal.pgen.1009026)
Supplement: Attachment — Submitted filename: Response_reviewers.pdf [file pgen.1009026.s014.pdf]

Reviewer's Responses to Questions

Comments to the Authors:

Please note here if the review is uploaded as an attachment.

Reviewer #1: Garner et al. describe the negative role of TOPLESS family members in SRFR1/SNC1-dependent immunity. Using plant dwarf morphology as a proxy to defense autoactivation, they performed epistasis analysis between srfr1 and tpr mutants, looking for enhancement or reversion of srfr1 phenotype. They demonstrated that trp2 and tpr3 loss-of-function mutant both enhance srfr1/snc1 stunting growth and defense gene expression. Whereas, TPR2 overexpression suppressed srfr1 and srfr1 tpr2 growth. They further showed that srfr1 tpr2 tpr3 phenotypes depend on SNC1. Coupled with gene expression analysis, the authors claimed that SRFR1 functions upstream of SNC1 transcription, while TPR2, on the other hand, may act downstream.

The manuscript is overall convincing and easy to follow. The genetic data is solid and clear; however, infection assays and mechanisms are lacking. To justify publication in PLOS Genetics, more characterization of the mutants and some mechanism are needed:

Major comments:

- Infection assays: why the authors did not perform a single infection assay for the mutants? Plant size and marker gene expression are helpful. But without bona fide pathogen challenges, it is insufficient to draw immune-related conclusions. It is reasonable bacterial infections are difficult to be done on dwarf mutants, but not wild-type like plants.

Agreed. Please see new S6 Fig and S7 Fig, and our responses to Reviewers 2 and 3.

- The role of TPR2/3 on SNC1 is unclear. At least some molecular mechanism should be provided.

Please see our summary response to reviewer 2.

Minor comments:

- It would be helpful to place parental lines in the same images:

Fig3A, srfr1 tpr3 double mutant

Fig4A, tpr2, tpr3 single mutant

The relative growth phenotypes of *srfr1-4* and higher order mutants are consistent, but the specific phenotypes are conditioned by the environment. Even in the same growth chambers plants vary slightly depending on the season (humid summer or dry winter). We therefore only show plants that were grown side-by-side at the same time, and analyzed replicated cohorts of plants separated in time to capture this variability. It is therefore not possible to add certain genotypes to a figure. Note that the *srfr1 tpr3* double mutant is shown in Fig. 1 in relation to Col-0, *srfr1-4* and *srfr1-4-tpr2-2*, and the *tpr2* and *tpr3* single mutants in Fig. 3 in relation to Col-0. We have added fresh weight measurements for all single mutants used in this study in new S1 Fig. See related new text lines 173-174.

- L140: to claim tpr1-2 is not a true knockout need to show T-DNA genotyping and RNA level.

All T-DNA lines and higher order mutants were genotyped. As explained in the manuscript, we focused on the novel phenotypes observed with mutations in *TPR2* and have added data

to show that both *tpr2-1* and *tpr2-2* are nulls (new S4 Fig and text lines 197-198). We have changed the wording to clarify that we are suggesting, not showing, that *tpr1-2* is not a complete knock-out (lines 181-183). We could remove data with *tpr1-2* without changing the main message of the manuscript. However, we think it is of value to the research community to show negative data as well to let researchers know that the *tpr1-2* T-DNA line is most likely not useful.

- L166, it is insufficient to claim tpr2 tpr3 act redundantly to enhance srfr1. Under autoimmune mutant background, this could also due to additive effects of independent pathways.
It appears to be accepted that TPL, TPR1 and TPR4 function redundantly following similar reasoning. However, we agree with the reviewer and have made changes in the text (lines 204 and 223). We thought redundancy is more likely given the lack of statistically significant stunting of *srfr1-4 tpr3-1* plants compared to *srfr1-4*, unlike *srfr1-4 tpr2-2* plants. This analysis though may miss a small effect in *srfr1-4 tpr3-1* plants and we agree that *srfr1-4 tpr2-2 tpr3-1* plants may display an additive effect.

- L168, may worthwhile performing infection assays on tpr2 tpr3 double mutants. Certain mutant with mild EDR phenotype may not exhibit obvious growth defects.
We agree with the reviewer that certain EDR plants do not show growth defects, as we previously showed for *srfr1-4 snc1-11* plants (Kim et al., 2010). It has been our observation that a slight increase of *SNC1* levels does not indicate a mild EDR phenotype, whereas a slight increase in *PR2* does. Please see bacterial growth curves in S6 Fig and S7 Fig, and text lines 229-230 and 267-268.

- L232: should it be "decrease" in SNC1 expression?
Yes, thank you for catching that! Now line 298.

- L240: no evidence supports TPR2 compete with TPR1
- Fig8C, in vitro assays are fine for protein interactions. I would like to see at least an additional in vivo IP to support the claim. Transient expression in tobacco would be sufficient.
- Fig9: Without additional data the model depicting competition between TPR2/SNC1 and TPR1/SNC1 cannot be made. These can also be tested via transient expression in tobacco.
Structural studies suggest that plant TPR proteins tetramerize (Ke et al, 2015, and Martin-Arevalillo et al, 2017, show tetramerization of TPL N-terminal domains). Our model for competition though was based on the information in Causier et al. (2012) that in yeast-two-hybrid assays TPR1 only interacts with itself and TPR4 with TPR1 and itself, but not TPR2 or TPR3. Transient expression of *SNC1* or of the TIR domain leads to HR in *Nicotiana benthamiana* and even at earlier time points to protein degradation. In addition, a co-IP does not give information on whether the interaction is direct. We therefore performed in vitro pull-downs, which for TPR1 have been shown to be biologically relevant (Zhu et al, 2010). Since both TPR1 (Zhu et al, 2010) and TPR2 (this manuscript) directly interact with the SNC1 TIR domain we did not think the proposition of competition is far-fetched and provides a parsimonious potential mechanism for the observed phenotypes.

We agree of course that more information is always desirable, and had experiments in the pipeline when research was ramped down in spring. Encouraged by the reviewers we first attempted to test competition in vitro, because relative degrees of protein interactions assayed by transient expression in *N. benthaiana* are difficult to quantify because of inconsistent protein levels in different samples, even from the same leaf. For unknown reasons though TPR1 did not express well in *E. coli* in our hands.
We therefore proceeded with co-IPs in *N. benthamiana eds1-1* plants. Surprisingly, we observed TPR1-TPR2 interactions in targeted TPR1-TPR2 co-IPs as a follow-up to SNC1 IPs that co-precipitated both TPR1 and TPR2. We do not feel justified in evaluating whether SNC1-TPR2 interactions are actually increased in the presence of TPR1 given the limitations of transient expression. If anything though this would indicate the preponderance of SNC1-TPR1-TPR2 heteromers. This would require detailed follow-up studies that we believe are beyond the scope of this manuscript. Taken together, we can propose that TPR2 by complexing with TPR1 alters the function of heteromeric SNC1-TPR complexes or titrates the availability of TPR1 for interaction with SNC1. We added the new co-IPs as Fig 9, and updated the model (now Fig 10) and text (Abstract lines 68-72, Results lines 306-317, Discussion lines 428-434).

Reviewer #2: The work is beautiful and the genetic data to reveal the interaction between SRFR1 and TPR2 and TPR3 is clearly present. However, about the function of TPR2 and TPR3 in negatively regulating plant autoimmunity, the conclusions are not that convincing, and the proposed mechanisms are a little speculative. Additive experiments are recommended to confirm the conclusions and strengthen the hypothesis.
Major
1.The authors claim a novel role of TPR2 and TPR3 in suppressing plant autoimmunity, pathogen assays are recommended to proof such a statement. PstDC3000 (virulent) or Pst DC3000 AvrRps4 (avirulent) strains could be used to test the srfr1-4 tpr2-2 and srfr1-4 tpr2-2 tpr3-1 mutants to provide further information on the effect of TPR2 and TPR3 on pathogen resistance.
Agreed, please see new S6 Fig and S7 Fig, and text lines 229-230 and 267-268.

2. The authors propose that TPR2 and to some degree TPR3 act downstream of SNC1 transcription by repressing expression of a positive regulator of SNC1 or activating a negative regulator. RNA-seq of srfr1-4, srfr1-4 tpr2-2, and srfr1-4 tpr2-2 tpr3-1 mutants are recommended, which could better explain the diverse function of these two TOPLESS members.
We agree with this suggestion but think it is beyond the scope of this manuscript. Without detailed analysis and follow-up verification we suspect that a first-pass RNA-seq analysis would merely reflect the different degrees of immune activation already demonstrated by the growth phenotype and expression levels of marker genes. The focus of our work was to establish the functional relationship between SRFR1 and TPRs. Since they act additively, it would be valuable to compare *snc1-1 tpr1* and *snc1-1 tpr2* plants by RNA-Seq to more directly identify differences in gene regulation. We value your suggestion.

Minor
1. In figure 1, the letters that denote the significant differences are placed on top of the

figure, which is difference from the letter placements in other figures. It is better to be consistent.
Done, Fig 1.

2. In page 5, line 85 "PR genes" should be "PR genes", "PR" should be in italics.
3. In page 9, line 171 "Fig 4C and 4D" should be "Fig 4B and 4C".
Thank you for catching both errors. Now lines 100 and 205.

Reviewer #3: from attachment
The manuscript reports new results on genetic interactions between *SRFR1*, encoding a negative regulator of ETI mediated by RPS4, SNC1 and several other NLRs, and members of the *TOPLESS* (*TPL*) gene family (*TPR*) of transcriptional co-suppressors in Arabidopsis. While some members of the later family see to have a role in the activation of defense responses, new results presented in this paper suggested a role of the family members *TPR2* and *TPR3* to prevent high defense activity. Conceptually this study is based on the observation that mutations in *SRFR1* result in elevated levels of expression of the NLR gene *SNC1*, which in turn triggers autoimmunity associated with stunted growth. The authors created a set of double mutants combining the *srfr1-4* mutant (which exhibits stunted growth) with T-DNA insertion mutants of *TPL/TPR* family members. The growth reduction observed in *srfr1-4* was found to be further amplified in *srfr1-4/tpl2* double mutants and even further enhanced in the *srfr1-4/tpl2-2/tpl3-1* triple mutant. The increase in stunting in these mutants was found to be correlated with enhanced expression of the defense marker gene *PR2* and *SNC1*. A loss of function allele of *SNC1* suppressed the growth-related effects caused by *srfr1*, *tpl2* and *tpl3* mutations. This confirmed that the observed effects are dependent on *SNC1*. Consistent with the inferred role of *TPL2* and *TPL3* in limiting the extent of autoimmunity seen in *srfr1* mutants, overexpression of *TPL2* in the *srfr1* background reduced the *srfr1* growth retardation phenotype. While direct physical interaction between the SRFR1 and TPL2 or TPL3 proteins were not examined in this study, the authors presented *in vitro* interaction data showing possible binding of TPL2 to the SNC1 TIR domain. Effects observed in the tested mutants on *SNC1* were also related to the NLR gene RPP4, which, to a certain extent, is co-regulated with *SNC1*.

Overall I find the presented results of good quality and the paper well written. An interesting new detail has been identified in the unexpected role of *TPR2* (and likely *TPR3*) in limiting SNC1-dependent defense activation. However, no new mechanistic insights were provided and the scope of the paper is somewhat limited. The conclusions are certainly interesting for audiences interested in molecular plant microbe interactions.

I have the following concerns/suggestions.

Major points:
1) Figure1: They state that the *tpr1-2* allele is not a true knockout. What is this conclusion based on? Either take results with this allele out of the figure or use a true knockout allele. Otherwise no clear conclusions can be drawn based on these results. How about the other T-DNA mutants of members of TOPLESS family? Are they true knockout alleles?
Please see our response to reviewer 1 and new S4 Fig.

2) The authors proposed a model for TPR2/TPR3 and SRFR1 functions in SNC1-mediated autoimmunity. However, only the morphological phenotype of plants and *SNC1* or *PR2* gene expression data were presented. Is this morphological phenotype correlated with a disease/resistance phenotype? I am aware that *Pseudomonas syringae* assays with severe dwarf mutants are not trivial. However, I think if autoimmunity-related effects are claimed, defense assays with a virulent pathogen strain (such as Pst DC3000) must be included.
We agree with the reviewer that bacterial assays with severely stunted plants are very difficult, and perhaps difficult to interpret with altered leaf thickness etc. In addition, it is very well established that the parameters we describe here (degree of stunting, *PR2* levels) correlate very well with plant resistance. In response to reviewers though we provide bacterial growth data for genotypes that are not stunted (new S6 Fig and S7 Fig). For the latter related to main Fig. 6, note that a slight increase in *PR2* expression correlated with increased resistance.

Minor points:
3) Early study showed *srfr1* morphological phenotype to be temperature dependent. How is this with *tpr2*?
As expected with a *SNC1*-dependent phenotype, higher temperatures relieve the stunted phenotype of *srfr1-4 tpr* lines (new S3 Fig, text lines 168-171).

4) Line 205, Fig 5C should be Fig 6C
Done, now line 244.

Please note that we also made changes to the following figures. We noticed small errors while compiling all data in the submitted Excel spreadsheet. These errors do not change the conclusions in the manuscript.
1. Fig. 7B: Col-0 and *snc1-1* are not significantly different. We therefore placed a letter "a" above *snc1-1 tpr2-2* and "b" above the other two plants.
2. The labeling of the second and third plant genotypes in Fig. 8B were switched. It now correctly reads from the left: Col-0, *eds1-2*, and *eds1-2 tpr2-2*.
