# Supplementary material for: Opposing functions of the plant TOPLESS gene family during SNC1-mediated autoimmunity
Source: PLoS Genet. 2021 Feb 23;17(2):e1009026. doi: 10.1371/journal.pgen.1009026 (PMC7935258; doi:10.1371/journal.pgen.1009026)
Supplement: Attachment — Submitted filename: Response to reviewers.pdf [file pgen.1009026.s015.pdf]

Response to reviewers

Thank you for the positive reviews and strengthening our manuscript.

Reviewer #1: The authors have addressed most of my concerns. This reviewer has a couple of final suggestions:

1. As a dominant-negative mutant of topless tpl-1 is lethal, it is predicted that knocking out all five TPL/TPR related genes would yield a lethal phenotype. Therefor the whole TPL family must be redundant in many aspects. This may explain the weak phenotypes of the mutants the authors describe. The new interaction data between TPR1 and TPR3 can also support such redundancy since they are exchangeable in the complex. Some discussion should be added regarding this, so the readers would bear in mind the diverse, and sometimes even opposite roles of these repressor proteins in regulating plant biology. An alternative explanation for the story can be that the TPL family proteins all serve both positive and negative roles, the previous 3 more positive, while 2/3 more negative. I would also suggest the authors to include such alternative model in their model figure.

We thank the reviewer for these insightful comments. We have attempted to address them by revising the discussion of our model (see bottom page 14 - page 15 and pages 17-18). We felt our model figure is already quite complex, and that adding more elements would make it less intelligible. We hope the combination of figure and more detailed discussion presents a useful framework for further experiments.

Please note that we updated Figure 10 in one thing though. Reexamining everything we noticed that the thick curved green arrow on the right side of the figure had been shifted too far left in the previous version.

2. Some main figures are quite thin, and there are too many figures/sup figures. I would suggest the authors to reduce the number of figures below 7, combining some simples ones but include some of the important supplementary figures in main.

We agree that the amount of data in our figures varies. We designed them for clarity more than for uniform density. We appreciate PLoS' guidelines that the number of figures is not limited and attempted to group data according to message and to clearly separate different mutant combinations, with confirmatory data in supplemental figures. We prefer to keep the figures as they are.

Reviewer #2: In the revised manuscript, the authors added data on planta bacterial growth assays. Their conclusions about the role of TPR2 and TPR3 in suppressing plant autoimmunity are more solid and convincing now with the addition of the new generated data. Most of my previous concerns have been well addressed.

One of my previous concerns was not entirely addressed: although the authors showed that TPR2 directly interacts with SNC1, the mechanism on how this interaction represses negative regulators of immunity is still not quite clear.

We agree that more work is required to fully understand TPR2's role in regulating SNC1.

Since the main focus of this manuscript is to establish the functional relationship between

SRFR1 and TPR, it is acceptable that the authors did not look into the detailed differences in gene regulation by RNA-Seq in this manuscript.

Reviewer #3: My concerns have been sufficiently addressed.
