# Supplementary material for: Opposing functions of the plant TOPLESS gene family during SNC1-mediated autoimmunity
Source: PLoS Genet. 2021 Feb 23;17(2):e1009026. doi: 10.1371/journal.pgen.1009026 (PMC7935258; doi:10.1371/journal.pgen.1009026)
Supplement: Attachment — Submitted filename: PG-20-Gassmann.docx [file pgen.1009026.s013.docx]

The manuscript reports new results on genetic interactions between *SRFR1*, encoding a negative regulator of ETI mediated by RPS4, SNC1 and several other NLRs, and members of the *TOPLESS* (*TPL*) gene family (*TPR*) of transcriptional co-suppressors in Arabidopsis. While some members of the later family see to have a role in the activation of defense responses, new results presented in this paper suggested a role of the family members *TPR2* and *TPR3* to prevent high defense activity. Conceptually this study is based on the observation that mutations in *SRFR1* result in elevated levels of expression of the NLR gene *SNC1*, which in turn triggers autoimmunity associated with stunted growth. The authors created a set of double mutants combining the *srfr1-4* mutant (which exhibits stunted growth) with T-DNA insertion mutants of *TPL/TPR* family members. The growth reduction observed in *srfr1-4* was found to be further amplified in *srfr1-4/tpl2* double mutants and even further enhanced in the *srfr1-4/tpl2-2/tpl3-1* triple mutant. The increase in stunting in these mutants was found to be correlated with enhanced expression of the defense marker gene *PR2* and *SNC1*. A loss of function allele of *SNC1* suppressed the growth-related effects caused by *srfr1*, *tpl2* and *tpl3* mutations. This confirmed that the observed effects are dependent on *SNC1*. Consistent with the inferred role of *TPL2* and *TPL3* in limiting the extent of autoimmunity seen in *srfr1* mutants, overexpression of *TPL2* in the *srfr1* background reduced the *srfr1* growth retardation phenotype. While direct physical interaction between the SRFR1 and TPL2 or TPL3 proteins were not examined in this study, the authors presented *in vitro* interaction data showing possible binding of TPL2 to the SNC1 TIR domain. Effects observed in the tested mutants on *SNC1* were also related to the NLR gene RPP4, which, to a certain extent, is co-regulated with *SNC1*.

Overall I find the presented results of good quality and the paper well written. An interesting new detail has been identified in the unexpected role of *TPR2* (and likely *TPR3*) in limiting SNC1-dependent defense activation. However, no new mechanistic insights were provided and the scope of the paper is somewhat limited. The conclusions are certainly interesting for audiences interested in molecular plant microbe interactions.

I have the following concerns/suggestions.

Major points:

1) Figure1: They state that the *tpr1-2* allele is not a true knockout. What is this conclusion based on? Either take results with this allele out of the figure or use a true knockout allele. Otherwise no clear conclusions can be drawn based on these results. How about the other T-DNA mutants of members of TOPLESS family? Are they true knockout alleles?

2) The authors proposed a model for TPR2/TPR3 and SRFR1 functions in SNC1-mediated autoimmunity. However, only the morphological phenotype of plants and *SNC1* or *PR2* gene expression data were presented. Is this morphological phenotype correlated with a disease/resistance phenotype? I am aware that *Pseudomonas syringae* assays with severe dwarf mutants are not trivial. However, I think if autoimmunity-related effects are claimed, defense assays with a virulent pathogen strain (such as Pst DC3000) must be included.

Minor points:

3) Early study showed *srfr1* morphological phenotype to be temperature dependent. How is this with *tpr2*?

4) Line 205, Fig 5C should be Fig 6C
